# Hospital do Rim: 25 years as the global leader in kidney transplants

**DOI:** 10.1590/2175-8239-JBN-2024-0076en

**Published:** 2024-08-09

**Authors:** Maria Lúcia Vaz, Lúcio Requião-Moura, José Medina Pestana

**Affiliations:** 1 Fundação Oswaldo Ramos, Hospital do Rim, São Paulo, SP, Brazil.; 2 Universidade Federal de São Paulo, São Paulo, SP, Brazil.

Dear Editor,

In 2023, the Hospital do Rim celebrated its 25^th^ anniversary with the expressive mark of 19,000 transplants performed since its creation in September 1998. At the same time, a significant milestone was reached this year: 1,111 kidney transplants, 7% of which were in pediatric recipients, 16% in the elderly, and 82% from deceased donors. Alongside the center has significantly improved patient outcomes: the one-year death-non-censored graft survival rate has increased from 77% in the 1990s to 93% last year for deceased donor recipients and from 94% to 99% for living donor recipients; and the incidence of acute rejection in the first year has steadily decreased, reaching 6% to 10% in the last few years. As part of the efforts to generate high quality information, each patient’s data is included in the extensive database of the Collaborative Transplant Study – CTS, which is based at the Heidelberg University in Germany.

The large-scale transplant program is based on concepts used in industrial assembly line production, including meticulous planning, structured and sequential workstations, and systematic performance monitoring^1^. Five individual workstations with a highly specialized workforce are crucial for this process. The first workstation is the organ procurement organization, serving an area with 75 hospitals and a population of 7 million in the state of São Paulo, as one of 10 organ procurement organizations serving over 1,120 hospitals with a population of 45 million. In the second unit, patients referred for kidney transplantation throughout the country are evaluated, selected and prepared by healthcare workers. The third station manages selected patients’ admissions, transplant surgeries, and immediate post-operative care until discharge. The fourth station is responsible for the long-term follow-up care of outpatients after transplantation. Finally, the central registry and research station provides timely data on results and quality standards. These interconnected stations are designed to ensure that every patient and their family members receive qualified care.

The pre-transplant outpatient clinic is a hub of multidisciplinary expertise and follows a streamlined clinical protocol for the evaluation of transplant candidates. It ensures all necessary examinations and tests are carried out, usually on the same day for deceased donor candidates, allowing immediate listing, which currently has 12,345 patients. The post-transplant outpatient clinic is also a multidisciplinary center responsible for 11,875 transplant recipients. In recent years, the integration of a telehealth service has improved outpatient care by offering teleconsultations for outpatients and interprofessional teleconsultations for patients treated in other institutions.

The large-scale environment of the Hospital do Rim provides an ideal setting for medical training and a wide spectrum of research. The center has become a training ground for 2,521 healthcare professionals, including 2,385 from Brazil, 88 from Latin America and 48 from Europe, Africa, and Asia. The hospital has a well-organized research and education division that focuses on both academic and clinical research. In the academic sector, 75 students completed their master’s degrees and 24 students have earned their PhDs. The center has participated in 182 trials, 60 of which are currently ongoing. Since 1998, Hospital do Rim staff and researchers have published 450 peer-reviewed international papers, including 51 on the impact of COVID-19 on the transplanted population.

The Hospital do Rim has gained international recognition as a leading center for kidney transplantation^2^. In the field of organ donation, the center has always been committed to the long-term well-being of living donors and has achieved remarkable success in optimizing the use of organs from deceased donors. A decade ago, the hospital innovated transplantation practices by introducing an immunological induction protocol that includes a single post-operative dose of 3.0 mg/kg thymoglobulin in the first 12 to 24 hours after transplantation^3^. This, combined with the strategic implementation of an unacceptable mismatch allocation system, has significantly reduced acute rejection rates in deceased donor recipients to consistently below 10%^4^. Additionally, the hospital has developed a comprehensive strategy to reduce the risk of cytomegalovirus infection using preemptive treatment^5^. Needless to say, the facility has been putting diversity and inclusion into practice long before its rise in society, as the majority of managers are women.

All of these legacies reflect the institutional mission to make excellence a habit, and success goes beyond mere statistics. The Hospital do Rim serves as a paradigm within Brazil’s public health system, demonstrating exceptional efficiency in the use of public resources and contributing to one of the most successful public policies in our country with the Brazilian Transplant Program, underscoring the commitment to improve kidney transplantation care and accessibility on a global scale.
